# It Is Time to Strengthen the Malaria Control Policy of the Democratic Republic of Congo and Include Schools and School-Age Children in Malaria Control Measures

**DOI:** 10.3390/pathogens11070729

**Published:** 2022-06-26

**Authors:** Sabin S. Nundu, Shirley V. Simpson, Hiroaki Arima, Jean-Jacques Muyembe, Toshihiro Mita, Steve Ahuka, Taro Yamamoto

**Affiliations:** 1 Institut National de Recherche Biomédicale, Kinshasa, Democratic Republic of the Congo; jjmuyembet@gmail.com (J.-J.M.); amstev04@yahoo.fr (S.A.); 2 Program for Nurturing Global Leaders in Tropical and Emerging Communicable Diseases, Graduate School of Biomedical Sciences, Nagasaki University, Nagasaki 852-8523, Japan; dweeny14@gmail.com (S.V.S.); taro.daichi.yamamoto@gmail.com (T.Y.); 3 Department of International Health and Medical Anthropology, Institute of Tropical Medicine, Nagasaki University, Nagasaki 852-8523, Japan; h.arima@nagasaki-u.ac.jp; 4 Department of Tropical Medicine and Parasitology, Faculty of Medicine, Juntendo University, Tokyo 113-8421, Japan; tmita@juntendo.ac.jp

**Keywords:** malaria, national malaria control program, school-age children, Democratic Republic Congo

## Abstract

Despite a decade of sustained malaria control, malaria remains a serious public health problem in the Democratic Republic of Congo (DRC). Children under five years of age and school-age children aged 5–15 years remain at high risk of symptomatic and asymptomatic malaria infections. The World Health Organization’s malaria control, elimination, and eradication recommendations are still only partially implemented in DRC. For better malaria control and eventual elimination, the integration of all individuals into the national malaria control programme will strengthen malaria control and elimination strategies in the country. Thus, inclusion of schools and school-age children in DRC malaria control interventions is needed.

## 1. Introduction

Malaria remains a major public health concern in sub-Saharan Africa (SSA), which in 2020 accounted for 96% of the estimated 241 million cases and 627,000 deaths globally [1]. Despite a decade of sustained malaria control and elimination strategies, the Democratic Republic of the Congo (DRC) had the highest malaria burden in the world after Nigeria, and in 2020 accounted for 12% of all estimated malaria cases and deaths worldwide [1]. Malaria is still the principal cause of morbidity and mortality in the country, with more than 25 million malaria cases and roughly 25,000 malaria deaths in 2020; of which children under five years of age accounted for 13 million malaria cases and 17,830 malaria deaths [2]. Malaria-related public health efforts in the country mainly focus on diagnosis with rapid diagnostic tests (RDTs), treatment with artemisinin-based combination therapy (ACT), and the distribution and use of insecticide-treated bed nets [2]. There are scant data available regarding countrywide malaria prevalence in school-age children, as they are not routinely included in household cluster surveys [3,4,5,6,7]. Children under five years of age and pregnant women are mainly targeted by national malaria surveys; including demographic health surveys (DHS), multiple indicator cluster surveys (MICS), the health management information system (HMIS), and district health information software 2 (DHIS). However, in 2010, 200 million school-age children between 5 and 15 years of age were estimated to be at risk of malaria in Africa, compared with 125 million children under five years of age [8]; thereby highlighting a shortfall in the structure of malaria screening. The school-age group is at high risk for both asymptomatic and symptomatic malaria infections [9,10,11,12,13]. Roughly 97% of the population of DRC lives in stable malaria transmission zones, in which transmission occurs for 8 to 12 months of the year [4]. It has been shown that in high transmission sites, symptomatic malaria is common in children under five years old; whereas asymptomatic infections generally occur in school-age children and adults, who have built immunity against the disease in response to repeated exposure [14,15,16,17]. *Plasmodium falciparum* is the most frequent *Plasmodium* species, and its prevalence peaks among school-age children [18,19,20,21,22,23,24], which in many areas can be over 50% [8,13,25]. School-age children have been shown to harbor more *P. falciparum* than children under five years of age and adult individuals in SSA including in DRC [7,13,18,20,22,23,26]. A prospective longitudinal study conducted in Kinshasa across 7 sites, 242 households, and 1591 individuals showed that the prevalence of infection peaked in school-age children and that they harbored more malaria parasites than other age groups [18]. School-age children are also a reservoir of parasite sexual stages, and are thereby an important source of human-to-mosquito malaria transmission [27,28], thus posing a major challenge for malaria control and elimination efforts [29,30,31]. Even though school-age children rarely develop complicated forms of malaria, chronic infection among this group is a contributor to pathology, such as anaemia. Chronic infection may have consequences for neuro-cognitive development and educational achievement, including increased absenteeism, poor school performance, and cognitive disorders [32,33,34,35,36,37]. It has been shown that malaria was responsible for about 13–50% of all school absenteeism among school-age children in endemic settings [38]. Thus, school-age children are neglected and are the group least likely to profit from malaria interventions in the country [3,4,5,6,39,40]. Moreover, they are the group least protected with insecticide-treated nets (ITNs), as the vast majority of ITNs are distributed through the expanded program for immunization (children under five years of age) and antenatal care (pregnant women) [2,4], and rarely in schools [2]. School-age children may receive ITNs during mass campaigns every three years; but when the number of mosquito nets in a household is insufficient, they are given lower priority for their use compared with children under five years of age and pregnant women [2]. The World Health Organization (WHO) recommendations regarding preventive chemotherapy do not target school-age children; rather, they include intermittent preventive treatment of malaria in infants (IPTi) for children under 12 months living in high-transmission areas of Africa, intermittent preventive treatment of malaria in pregnancy (IPTp) for pregnant women living in moderate-to-high transmission areas, and seasonal malaria chemoprevention (SMC) for preschool children living in areas of the Sahel region of Africa [1]. 

We argue that malaria control and interventions must target all members of the community, including school-age children, to promote health and social equity benefits [41], in terms of access to malaria case management and prevention useful for malaria control and elimination.

Here, we review the DRC malaria control policy and show the necessity of policy improvement and the inclusion of schools and school-age children in the country’s malaria control and elimination strategies.

## 2. Current DRC Malaria Control Policy

DRC continues to explore mechanisms and strategies for malaria control and prevention which will significantly lower the transmission of the disease and have a positive impact on malaria elimination in the country. Currently, intervention measures recommended by WHO are partially implemented under the umbrella of the national malaria control program (NMCP). This includes, in brief, (i) malaria case management which involves giving antimalarial drugs only after a positive confirmatory RDT test and/or microscopy; (ii) vector control with ITNs for prevention of malaria which mostly targets higher risk groups, including children under five years of age and pregnant women, while indoor residual spraying (IRS) is included in the DRC malaria control policy but has not yet been implemented [1,2,4], except in some mining and private sectors [42,43]; and (iii) IPTp is given to pregnant women, while IPTi is listed in the DRC malaria policy but has not been implemented for infants [2,4].

### 2.1. Malaria Case Management

Based on the DRC malaria guidelines, all suspected cases of malaria must be tested with RDTs before starting treatment; and in the cases of negative results, other causes of fever are explored [2]. Microscopy and/or RDTs are the current routine diagnostic tests used for the confirmation of malaria-suspected cases prior to antimalarial treatment [2,4], in part to prevent drug resistance arising due to an overuse of drugs after erroneous presumptive treatments [44]. Although microscopic examination is regarded as the gold standard diagnostic tool, its use is challenging, especially in rural and semi-urban settings where there is a lack of experienced microscopists, equipment, reagents, and electricity, and, in the case of poor blood film preparation and when the parasitemia is low [45,46,47,48,49,50]. Thus, RDTs are mostly used as an alternative for quick and accurate diagnosis [51,52,53,54]. The DRC national malaria case management guidelines recommend the use of RDTs to test all suspected malaria cases at a health facility, community care site or licensed pharmacy; while microscopy is limited to suspected cases of treatment failure or any case of severe malaria to monitor parasite clearance. It is also used for parasite species identification. Microscopy can only be performed in facilities with appropriate equipment and trained microscopists; and this is usually limited to the referral health facility level [2,4].

The sensitivity and specificity of both microscopy and RDTs are dependent on threshold parasitaemia levels; and in the case of low parasitaemia, microscopic examination (less than 50–100 parasites per μL blood) and RDTs (less than 200 parasites per μL blood) may carry the risk of false negative results, compromising treatment for true cases [52,55,56,57,58,59,60]. 

Although RDTs and ACT are typically free of charge to patients, there is a risk of stockouts as almost all drugs, ITNs, and RDTs are provided by the Global Fund and the U.S. President’s Malaria Initiative (PMI) [2], and the DRC government does not contribute to the stocks. This situation may decrease the universal availability for patient diagnosis and treatment in health care facilities, and RDT stockouts may increase presumptive treatment of unconfirmed cases leading to treatment misuse. To address this issue, PMI recommends supplying health zones with six months of stock to mitigate stockouts during the period of inaccessibility. Additional stocks should be held at the health zone level to avoid overloading storage at health facilities [2]. Presumptive treatment of unconfirmed/negative cases is managed according to the guidelines of the integrated management of childhood illness, especially for children under five years of age with suspicion of severe malaria [2,4]. 

The strengthening of malaria case management requires the promotion of community case management of malaria (CCMm) as recommended by the WHO, to increase the number of patients with access to RDTs and ACT, especially for remote and marginalized populations [61,62,63]. Research conducted in other countries [64,65,66,67,68,69,70], and in the DRC [58,59,60] following WHO strategies based on CCMm, have shown a significant positive effect of adherence to case management guidelines by improving access to RDTs and ACT. This follows the WHO strategy of ‘test, treat, and track’ (T3), which recommends evidence-based diagnostic testing, treatment, and surveillance [71]. 

Ideally, every suspected malaria case should be tested, treated, and tracked to minimize presumptive treatment and save ACT from misuse and the risk of the development of parasite resistance. However, case management only targets symptomatic individuals, and has limited ability to reduce the parasite reservoir harbored in asymptomatic parasite carriers [72]. To sustain malaria control and elimination strategies, it is important to also target asymptomatic individuals living in moderate and high transmission settings. It has been shown that systematic screening followed by treatment of asymptomatic individuals in high-transmission settings may sustain malaria control interventions and contribute to malaria elimination [73,74]. School-based malaria prevalence surveys (SMPS) are cost-effective and easier to conduct as compared with community-based malaria prevalence surveys (CMPS) [12,75]. Moreover, the malaria burden within school-age children is a reliable indicator of the prevalence and transmission intensity of the disease in a defined community [75], and SMPSs represent a smaller administrative unit to derive malaria infection estimates, relative transmission risk, and the impact of interventions [75,76]. An efficient SMPS may provide valuable information on intervention performance, which is useful for disease surveillance [75,77,78,79,80]. School health education may deliver suitable messages on the use of early detection of malaria and how to access prompt treatment [81], and a means to deliver ITNs that may benefit the community [81,82]. Schools are a community hub and can enhance community-wide malaria control by improving malaria awareness and prevention in both the children that they serve and the community [83]. Therefore, skills-based school health education can help promote a community-wide understanding of malaria and how to treat and prevent it [83]. It will additionally protect this underserved age group [78] by helping to integrate and control other tropical diseases that threaten them, such as soil-transmitted helminths, schistosomes, and filaria. Thus, it is important to enhance control measures for asymptomatic malaria parasite carriers to protect them against chronic infections leading to chronic anaemia, absenteeism, reduced school performance, and other complications [32,84,85]. 

### 2.2. Vector Control

ITNs are the only vector control widely implemented in DRC for the prevention of malaria, and with emphasis on children under five and pregnant women. From 2010 to 2018, the parameters of household ITN ownership, ITN use among children under five years of age and pregnant women increased from 30%, 38% and 43%, to 44%, 51%, and 52%, respectively [4]. However, the improvement of ITN coverage is still challenging due to the scarcity of mass distribution campaigns, which under DRC malaria policy now occur every three years [4]. In 2019, ITN coverage was estimated to be around 65% [1]. It is meaningful to monitor ITN replacement needs [86] every year after distribution. Additionally, ITN ownership is not usually correlated to ITN use and low bed net use may be associated with low awareness of malaria prevention [87], low education of mothers and other vulnerable individuals [40,86,88], inconvenience of net installation [89,90], and damaged or worn mosquito nets [91]. Thus, follow-up and evaluation of behavior change intervention focusing on malaria prevention, education, and promotion of the use of ITNs may increase the utilization of ITNs among underserved and vulnerable groups [40,87,88,92]. 

Although the distribution of ITNs targets the general population, the proportion of ITN use in school-age children is still quite less than children under five years of age and pregnant women [88,91,93,94,95]. There is an urgent need to promote a campaign of mass distribution of ITNs among school-age children through school-based or community-based interventions. The rationale is two-fold: to address the health welfare of this group, and because school-age children are a reservoir of gametocytes that mediate malaria transmission to mosquitoes [87,88,91,93,94,95]. Country reports have shown that the use of ITNs has reasonably reduced childhood morbidity and mortality [39,87,96] and the malaria burden among school-age children [43,86,97]. IRS is not yet officially implemented in DRC [4,42], but given the fact that the proportion of bed net ownership and use remains insufficient, the combination of ITNs and IRS may help to address malaria morbidity and mortality among susceptible groups. A study conducted in southern DRC has demonstrated the positive impact of the combination of strategies (ITNs, IRS, CCMm and CMPS) on reducing malaria prevalence among children (including school-age children) [43].

### 2.3. Preventive Chemotherapy

Among the three preventive chemotherapy strategies recommended by the WHO, only IPTp-Sulfadoxine/Pyrimethamine (IPTp-SP) is implemented in DRC for pregnant women during antenatal care visits to prevent morbidity and mortality, and adverse pregnancy outcomes including anemia, low birth weight, and stillbirths [4]. The WHO recommends a minimum of three doses of IPTp (IPTp3+) with universal coverage of at least 80% of pregnant women [1]. However, in DRC, IPTp3+ implementation is less than the universal coverage and the majority of women attending at least four antenatal care visits receive less than three doses of IPTp [1,98]. Despite the increase in SP *falciparum* resistance in DRC [99,100,101,102,103,104], IPTp is still used for malaria prevention during pregnancy and protection against maternal anemia and low birthweight; especially when it is given in three or more doses [101], as shown in other countries [105,106,107,108,109]. 

The optimization of IPTp coverage will be cost effective for the most beneficial protection of pregnancy outcomes [108]. Nevertheless, infants are not covered by preventive chemotherapy strategies (IPTi) as recommended by the WHO. Preventive chemotherapy would also help children under five years of age (IPTc) who are more at risk of malaria morbidity and mortality, and among school-age children (IPTsc) who are a large reservoir of asymptomatic carriers for disease transmission. Reports have shown that IPTc, given every four months combined with timely treatment of febrile malaria illness, has significantly reduced malaria-related childhood morbidity and mortality [110,111,112,113,114]. Similarly, IPTsc when given every four or three months has provided substantial protection against malaria morbidity and anemia, as well as a reduction in school absenteeism and increased school performance [32,115,116,117]. 

However, SP resistance has spread globally, probably due to SP overuse, and in part because of improper self-medication [118]. SP self-medication is still widely used for treatment of uncomplicated malaria [119,120,121,122], and addressing its misuse and overuse will help to delay the increase in resistance. There is a need to continuously sensitize and educate women on the benefits of malaria prevention, including IPTp during pregnancy; and to educate community members, including community healthcare and private health professionals, about the guideline of malaria case management and malaria prevention [119,123,124,125,126,127]. To minimize the risk of SP resistance, SP may be used in combination with other non-artemisinin derivatives used as first-line treatment to protect ACT resistance [32,114,115,116,128,129].

Adherence to malaria preventive measures requires the promotion of social and behavioral change through education and training to increase the awareness and knowledge of populations such as parents (especially mothers), caregivers, teachers, and healthcare professionals, concerning malaria transmission risks, case management and prevention. Another malaria chemoprevention strategy is post hospital discharge administration of ACT to children after recovering from severe anemia [130,131]. This intervention has been shown to have a positive impact in Malawi, reducing by 31% the composite endpoints of death, severe anemia, or severe malaria when given one and two months after discharge of children under five years of age from hospitals [132]. In Kenya and Uganda, the strategy reduced all-cause readmission or death by 35% when given two, six, and 10 weeks after discharge [133]. For integration measures, it has been shown that the combination of vector control and preventive chemotherapy will reinforce malaria prevention strategies [134,135].

## 3. Conclusions

In the DRC, although school-age children are at a lower risk for the severe forms of malaria and mortality, they are at risk of symptomatic and asymptomatic malaria infections. However, they are neglected and are the group least likely to benefit from malaria interventions in the country. There is an urgent need to integrate school-age children into the national malaria control interventions including malaria DHS. Malaria control and interventions should target all members of the community, including school-age children to promote health and social equity benefits. DRC malaria control policy should also promote CCMm, SMPS and CMPS to strengthen malaria case management, vector control and chemoprevention strategies useful for malaria control and elimination.

## Data Availability

Not applicable.
